# Vitelline Vein Varix Diagnosed Postnatally Following a Prenatal Diagnosis of Umbilical Vein Varix: A Case Report and Review of the Literature

**DOI:** 10.7759/cureus.105019

**Published:** 2026-03-11

**Authors:** Reika Kanazawa, Ruriko Oyama, Keisuke Fukui, Takuya Aoki

**Affiliations:** 1 Obstetrics and Gynecology, Keiseikai Hospital, Osaka, JPN; 2 Obstetrics and Gynecology, Kobe City Medical Center General Hospital, Kobe, JPN; 3 Pediatric Surgery, Hyogo Prefectural Kobe Children’s Hospital, Kobe, JPN

**Keywords:** fetal intra-abdominal cystic lesion, neonatal vascular abnormality, portal vein thrombosis, umbilical vein varix, vitelline vein varix

## Abstract

Vitelline vein varices are rare vascular abnormalities. Differentiating them from umbilical vein varices during the prenatal period is often challenging; however, thrombus formation can occur in the early postnatal period, potentially leading to portal vein thrombosis, extrahepatic portal vein obstruction, and portal hypertension. Therefore, early diagnosis and appropriate therapeutic intervention are crucial for these patients.

We present a case that was initially diagnosed in utero as an umbilical vein varix but was subsequently confirmed after birth to be a vitelline vein varix complicated by portal vein thrombosis, which necessitated surgical intervention. The patient was a 35-year-old woman (gravida 2, para 1). At 26 weeks of gestation, a 15 mm cystic lesion with blood flow was identified within the fetal abdominal cavity and was initially thought to represent an umbilical vein varix. The patient was referred to our hospital at 29 weeks of gestation. During follow-up, the lesion increased in size to approximately 20 mm, and there was no evidence of thrombus formation. Fetal growth was appropriate for gestational age. The patient was admitted at 31 weeks of gestation. Due to her history of a previous cesarean delivery, an elective cesarean section was performed at 36 weeks, resulting in the delivery of a 2,744 g female infant with favorable Apgar scores.

On postnatal day 11, abdominal ultrasonography revealed a thrombus extending from the main portal vein into the intrahepatic branches. Contrast-enhanced CT on day 21 demonstrated a varix communicating with the superior mesenteric vein, which led to the definitive diagnosis of a vitelline vein varix. Subsequently, anticoagulation therapy with heparin sodium was initiated on postnatal day 25, followed by resection of the varix and thrombectomy on postnatal day 26. The postoperative course was uneventful. This report underscores the importance of considering vitelline vein varices in the differential diagnosis of fetal intra-abdominal cystic lesions with blood flow. It also emphasizes the need for thorough prenatal evaluation and a multidisciplinary approach to perinatal management.

## Introduction

Vitelline vein varix is a relatively recently recognized condition that was first reported in 2007 [1]. This condition refers to a dilated venous varix originating from the vitelline vein identified outside the fetal liver. To date, only 15 cases have been reported, indicating that it is an extremely rare entity. During the fetal period, differentiation from an umbilical vein varix is often challenging, and in many cases, the definitive diagnosis is established only after birth. This condition carries a high risk of early postnatal thrombus formation within the varix, which may progress to portal vein thrombosis and portal hypertension. Among the 15 previously reported cases, thrombus formation was observed in 12, and 10 of these required surgical intervention [1-5]. Although an umbilical vein varix also carries a risk of thrombosis, reported cases with actual thrombus formation are exceedingly rare.

The fetal venous system begins to develop at approximately five weeks’ gestation. The vitelline veins are essential vessels that return blood from the yolk sac to the fetal heart and subsequently form the portal venous system through selective fusion and regression [5]. Vitelline vein varix is considered a vascular developmental anomaly resulting from abnormal regression during this process. Furthermore, it has been reported that thrombi that form during the physiological postnatal regression of the umbilical vein may extend into the portal venous system, potentially resulting in portal vein thrombosis and portal hypertension within the first 10 days of life [6].

Early initiation of anticoagulation therapy, such as heparin sodium, is recommended after birth. When thrombus formation is identified, prompt surgical intervention, including varix resection, should be considered before progression to portal vein occlusion or dilatation occurs [1,3]. Therefore, close multidisciplinary collaboration among obstetricians, neonatologists, and pediatric surgeons is essential for optimal perinatal management of this condition.

We present a case that was prenatally suspected to be an umbilical vein varix but was definitively diagnosed after birth as a vitelline vein varix complicated by thrombosis, requiring surgical treatment. Because vitelline vein varices may necessitate surgical intervention in the early neonatal period, this condition should be included in the differential diagnosis when a fetal intra-abdominal cystic lesion with blood flow is detected. Therefore, careful diagnosis and management are crucial. We discuss the key diagnostic features and management considerations of this condition and review the relevant literature.

## Case presentation

The patient was a 35-year-old woman (gravida 2, para 1) with a history of emergency cesarean delivery at 33 years of age for fetal head malrotation during labor. The current pregnancy was conceived naturally. At 20 weeks of gestation, a 15 mm cystic lesion was detected in the fetal abdominal cavity on routine ultrasonography. She was referred to a tertiary care center at 26 weeks of gestation, where the lesion was diagnosed as an umbilical vein varix. Because the lesion was associated with abnormal placental positioning, she was referred to our hospital at 29 weeks of gestation for perinatal management.

**Figure 1:**
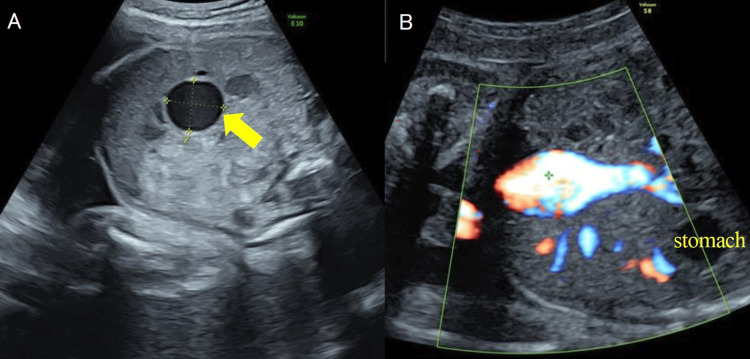
Fetal ultrasonography at 31 weeks of gestation (A) A cystic lesion measuring 22.1 x 19.8 mm was observed in the fetal abdominal cavity. (B) Color Doppler image demonstrating blood flow within the lesion, with no evident thrombus formation

During the two weeks following referral, the varix enlarged to approximately 20 mm; however, no clear evidence of intravariceal thrombus was observed (Figure 1), and fetal growth was consistent with gestational age. The patient was admitted for inpatient observation at 31 weeks of gestation. After admission, follow-up included abdominal ultrasonography twice weekly and fetal heart rate monitoring three times daily. The varix remained stable at approximately 20 mm, with no signs of thrombus formation. Doppler evaluation revealed normal blood flow in the umbilical artery, middle cerebral artery, and ductus venosus, with no findings suggestive of fetal heart failure; however, visualization of the ductus venosus was technically challenging.

At 36 weeks of gestation, an elective cesarean section was performed due to a previous cesarean delivery. A female infant weighing 2,744 g was delivered, with Apgar scores of 9 and 10 at one and five minutes, respectively. Postnatal abdominal ultrasonography performed on day zero revealed no apparent cystic lesions in the abdominal cavity. The neonatal course was uneventful, apart from physiological jaundice, and both the mother and infant were discharged on postnatal day six.

**Figure 2:**
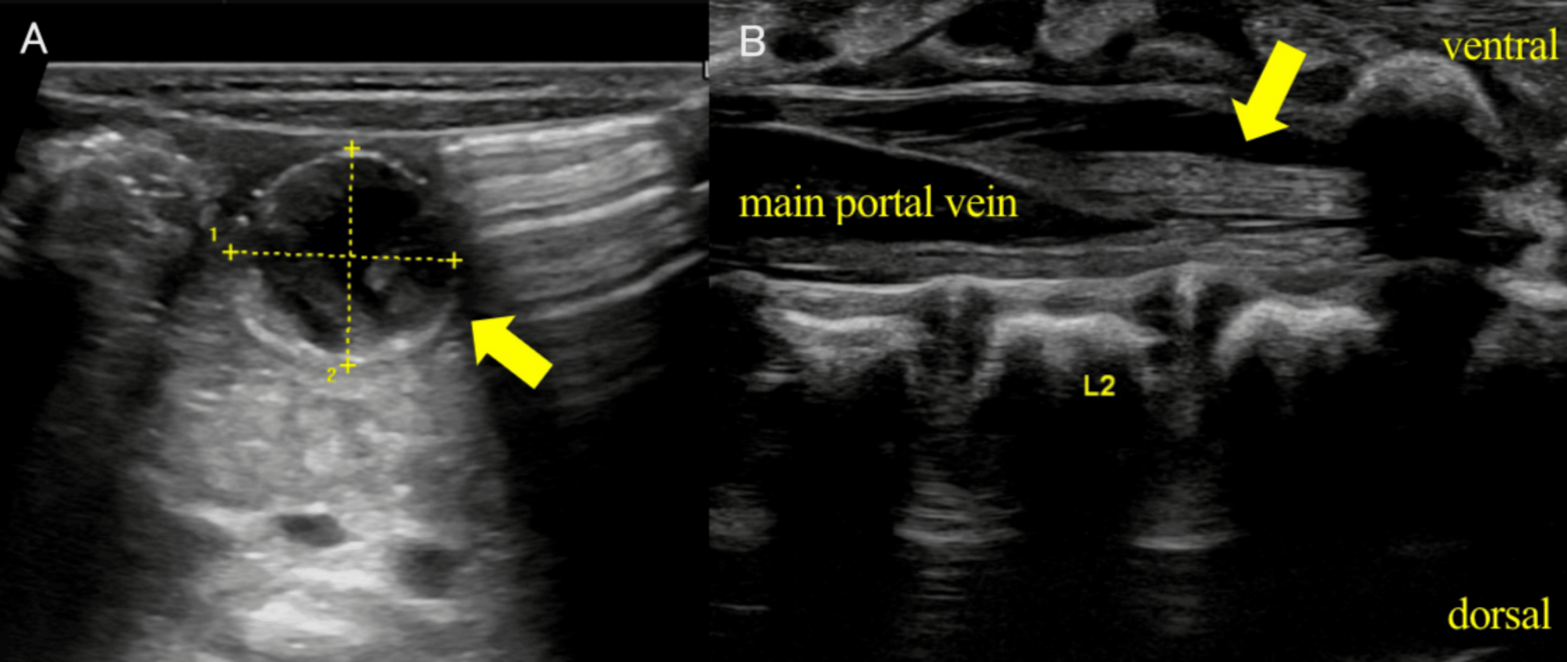
Neonatal abdominal ultrasonography on postnatal day 11 (A) A yolk vein varicosity measuring 15.1 x 14.7 mm with thrombus formation. (B) Thrombi extending from the main portal vein into the intrahepatic branches

**Figure 3:**
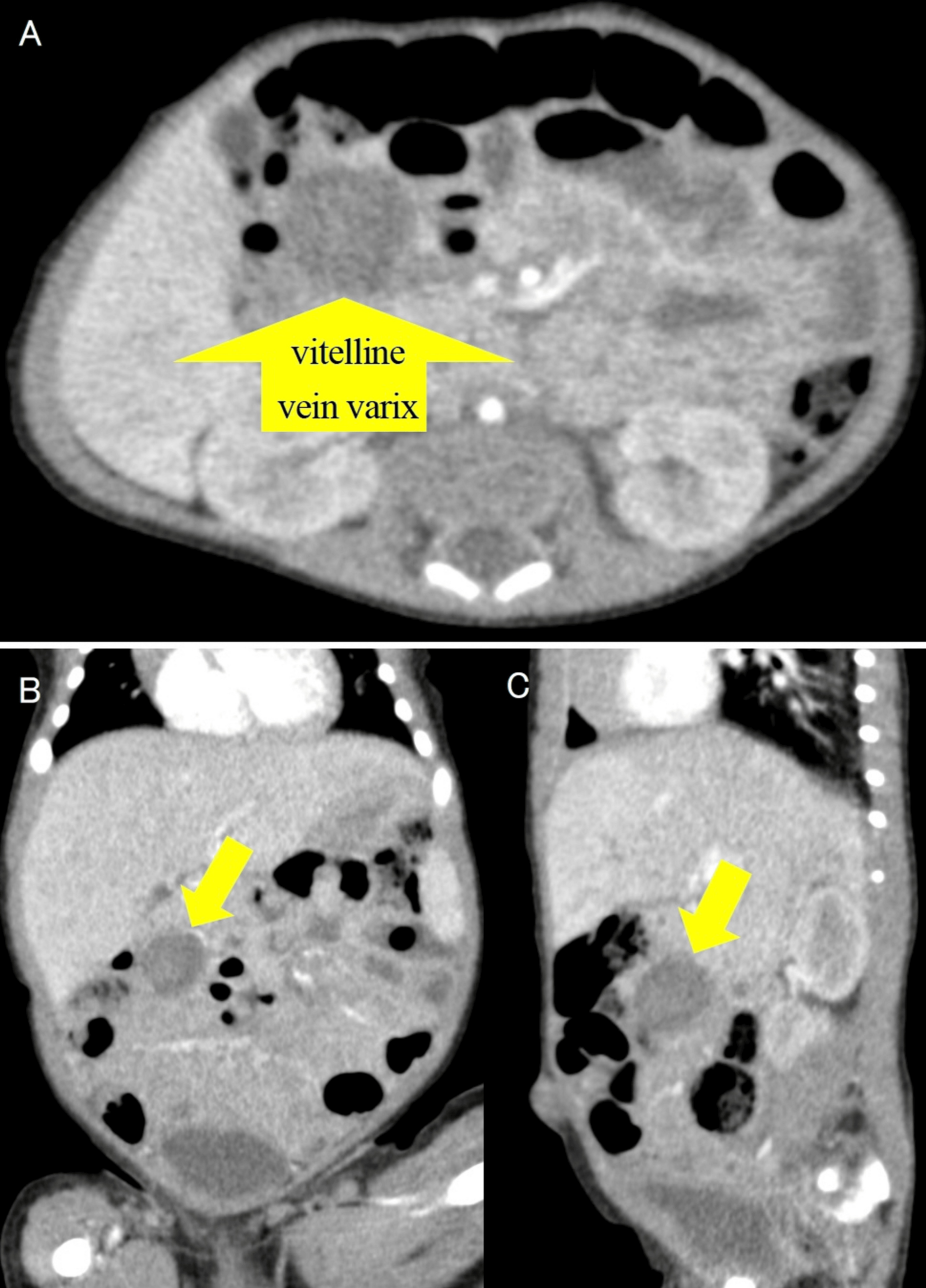
Contrast-enhanced CT on postnatal day 21 (A) Axial view. (B) Coronal view. (C) Sagittal view CT: computed tomography

On day 11 of life, the patient was referred to a tertiary pediatric perinatal center for evaluation. Screening abdominal ultrasonography revealed a thrombosed venous varix with thrombi extending from the main portal vein to the umbilical vein (Figure 2). Although an umbilical vein varix was initially suspected, partial thrombotic occlusion of the portal vein was identified, and further evaluation with an additional imaging modality was considered necessary. Contrast-enhanced CT was performed on day 21 of life, demonstrating a varix continuous with the superior mesenteric vein, leading to a definitive diagnosis of vitelline vein varix (Figure 3). Because an intravariceal thrombus was present and there was a risk of extrahepatic portal vein obstruction, early resection was deemed preferable. Anticoagulation therapy with heparin sodium was initiated on day 25 of life, and varix resection with thrombectomy was performed on day 26.

**Figure 4:**
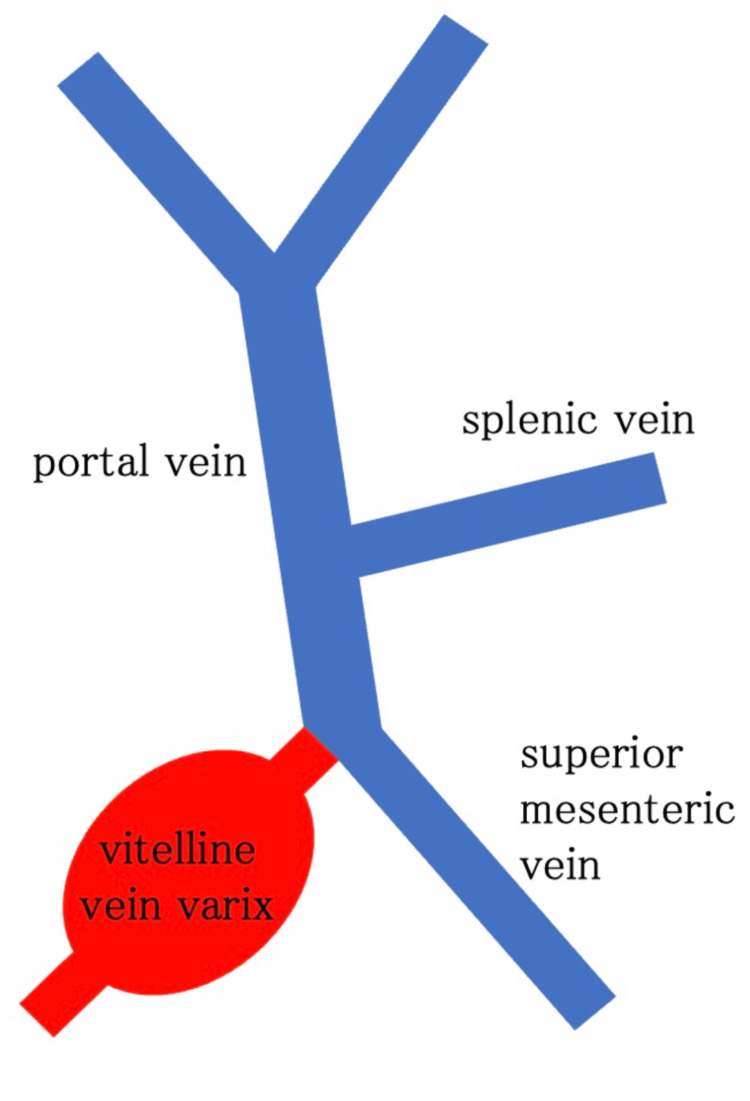
Intraoperative schematic illustration showing the location of the varicosity and its continuity with the portal venous system Image created by the authors by using Inkscape

Intraoperatively, the varix was located on the right anterior aspect of the transverse mesocolon and was continuous with the superior mesenteric and splenic veins, forming the portal vein (Figure 4). A remnant vitelline vein extending from the umbilicus to the varix was also identified, and the round ligament of the liver was absent. Intraluminal coagulated blood and thrombi were observed, and the lesions were completely resected. The postoperative course was uneventful, and the patient was discharged on postoperative day three. The infant is currently undergoing outpatient follow-up with careful monitoring for portal hypertension secondary to portal vein thrombosis.

## Discussion

The differential diagnosis of fetal intra-abdominal cystic lesions includes gastrointestinal obstruction, choledochal cyst, renal cyst, ovarian tumor, and umbilical vein varix [5]. When a cystic lesion with detectable blood flow is identified, a vitelline vein varix should also be considered in addition to an umbilical vein varix. Vitelline vein varix is extremely rare, and no clear association with fetal structural anomalies or chromosomal abnormalities has been reported to date. In contrast, umbilical vein varix has been reported in some cases to be associated with fetal malformations and chromosomal abnormalities [7-9]. Considering its embryologic background, careful fetal morphological assessment and chromosomal evaluation are recommended in cases of vitelline vein varix.

Embryologically, the portal vein and the umbilical vein do not directly communicate. However, vitelline vein varix forms between the vitelline vein and the portal vein and drains into the portal vein near the confluence with the superior mesenteric vein, creating an apparent communication between the portal venous system and the umbilical region [1,5]. Therefore, a detailed evaluation of the perihepatic venous anatomy is essential for accurate diagnosis.

Vitelline vein varix is believed to arise from incomplete regression of the right vitelline vein, resulting in the persistence of an abnormal vascular connection with the left umbilical vein. This anomalous connection creates an abnormal blood flow pathway that does not follow the normal course of the umbilical vein [1,5]. The abnormal venous pathway observed in the present case can be explained by this mechanism. After birth, thrombi formed during the physiological regression of the umbilical vein may extend into the portal venous system via the vitelline vein varix. This mechanism may account for the early postnatal portal vein thrombosis observed in this case. Previous reports have similarly described early-onset portal vein thrombosis and subsequent portal hypertension developing through this process [6].

**Figure 5:**
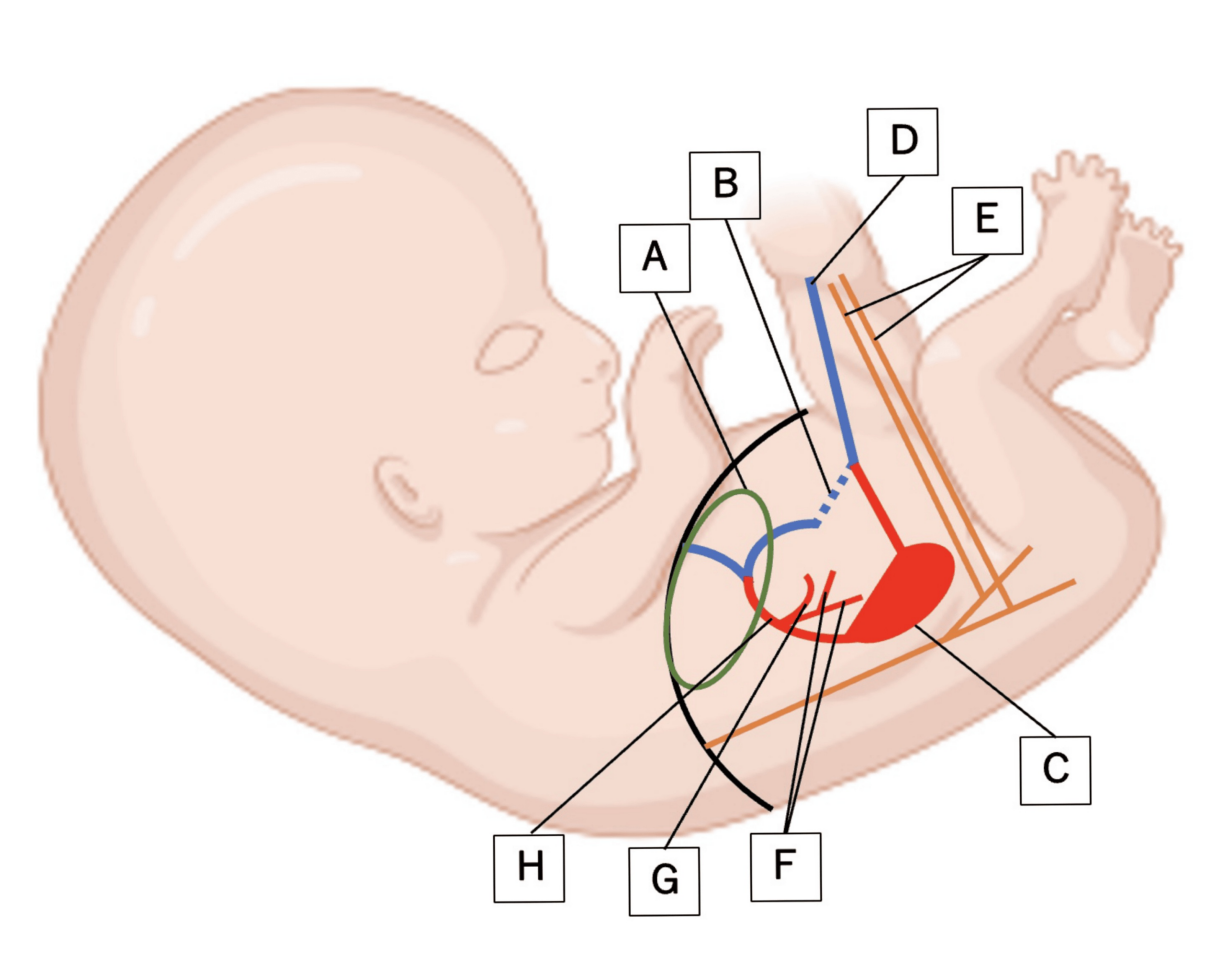
Schematic sagittal section of the fetus* ^*^Modified from [1] (A) Liver. (B) Normal course of the intra-abdominal umbilical vein. (C) Yolk vein varicosity. (D) Intrafunicular umbilical vein. (E) Intrafunicular umbilical arteries. (F) Superior mesenteric vein. (G) Splenic vein. (H) Portal vein Image created by the authors with BioRender.com and edited using Inkscape

Although prenatal differentiation between vitelline vein varix and umbilical vein varix is extremely challenging, previous reports and the present case suggest several practical diagnostic clues (Table 1) [5]. First, the timing of detection may provide useful information: vitelline vein varix tends to be identified relatively early, around 23-24 weeks of gestation, whereas umbilical vein varix is typically detected at 27-29 weeks [2]. Second, the venous course differs: umbilical vein varix generally courses cranially and ventrally from the gallbladder, whereas vitelline vein varix more often courses caudally and dorsally (Figure 5) [2,10]. Third, lesion size may aid differentiation: most umbilical vein varices measure less than 20 mm, whereas vitelline vein varices frequently exceed 20 mm [5,11,12].

**Table 1:** Summary of 15 reported cases of vitelline vein varix* ^*^Modified from [5] NR: not recorded; UVV: umbilical vein varix, VVV: Vitelline vein varix, DOL: days of life; HOL: hours of life, SMV: superior mesenteric vein; PT: portal vein thrombosis; PH: portal hypertension; CH: cavernous hemangioma at the hepatic hilum; EV: esophageal varices

Case	Year of report	Varix size				Initial diagnosis	Birth weight		Timing of heparin initiation	Thrombus		Operative findings		Clinical course and complications
		Gestational age at detection (weeks)	Size (mm)	Gestational age at detection (weeks)	Size (mm)									
							Weeks	g		Age at detection (DOL)	Location (DOL)			
												Days of life	Varix resection	
1	2007	24	21	32	36×54	UVV	34	2,100	NR	3	Intrahepatic portal vein(6)	9	Yes	PT, PH, CH
2	2008	25	24×22	NR	27	UVV	29	1,170	NR	No		0	Yes	
3	2008	NR	NR	NR	NR	UVV	Term	NR	NR	3	SMV confluence	6	Yes	Spontaneous thrombus resolution (2 weeks of age)
4	2010	23	15×7	33	27×13	UVV	34	2,606	NR	34 weeks of gestation	NR	No		No thrombus (6 months)
5	2012	24	NR	NR	NR	NR	35	NR	Yes	Yes	NR	NR	Varix ligation	
6	2013	24	21	32	54×36	UVV	34	2,100	No	3	Portal vein (6)	11	Yes	PH, CH
7	2013	20	25	30	48	UVV	37	2,200	No	1	Portal vein (6)	9	Yes	PH, CH, EV
8	2013	20	28	30	47	UVV	39	2,340	DOL6	1	Left portal vein (1)	9	Yes	Normal portal flow; no thrombus
9	2014	NR	NR	NR	NR	UVV	38	3,720	No	HOL5	Umbilical vein confluence	NR	Yes	
10	2014	22	NR	NR	NR	UVV	40	2,790	DOL5	2	Right portal vein (3), complete portal vein occlusion (4)	No		Complete portal vein occlusion with collaterals
11	2015	30	30	NR	NR	NR	36	2,320	No	1	Within the varix	3	Yes	
12	2018	23	33×25	34	33×31	VVV	34	2,400	No	Yes	Within the varix	NR	Yes	
13	2019	28	26×16	36	30×26	UVV	39	2,965	NR	3	Within the varix	NR	Yes	
14	2014	36	30×20	NR	NR	UVV or VVV	38	2,688	Immediately after birth	No		0	Yes	
15	2016	31	NR	33	22×14	UVV or VVV	36	2,430	Immediately after birth	No		0	Yes	

In the present case, the lesion was detected relatively early at 20 weeks of gestation, subsequently grew beyond 20 mm, and the ductus venosus was challenging to visualize, all of which are consistent with the characteristics of vitelline vein varix. These findings suggest that prenatal suspicion of this condition could potentially be raised using fetal imaging alone. However, because vitelline vein varix was not yet fully recognized as a disease entity at that time, it was not included in the differential diagnosis. A more detailed evaluation of the venous anatomy might have enabled prenatal recognition of this condition.

From a management perspective, this condition carries a high risk of early postnatal thrombosis. In the present case, thrombus formation was observed in the early neonatal period, requiring surgical treatment. During fetal assessment, not only lesion size but also morphology, internal echogenicity, and changes in the diameters of inflow and outflow vessels should be carefully evaluated, together with Doppler findings and signs of cardiac load. Enlargement of the varix and vascular dilatation may indicate hemodynamic impact, whereas increased internal echogenicity and vascular tortuosity or stenosis may represent early signs of thrombosis.

Currently, no established guidelines exist for the management of pregnancy and delivery in cases of vitelline vein varix, and consensus has not been reached even for the management of umbilical vein varix. Some reports recommend delivery at 34-36 weeks of gestation after confirming fetal lung maturity in cases of umbilical vein varix without thrombosis, whereas others support expectant management until term [5]. Therefore, management should be individualized based on detailed case evaluation. In the present case, no obvious cystic lesion was identified on abdominal ultrasonography on day zero of life. However, because a fetal intra-abdominal cystic lesion with blood flow had been detected prenatally, further postnatal evaluation and follow-up were considered necessary, and the patient was referred to a specialized pediatric center. At the time of evaluation at the pediatric center on day 11 of life, abdominal ultrasonography revealed a thrombosed venous varix and portal vein thrombosis, suggesting that thrombotic changes had already developed during the early neonatal period.

Loss of intravariceal blood flow and subsequent thrombosis in the early postnatal period may have made the cystic structure difficult to visualize. Therefore, the primary reason the lesion was not detected on day zero of life was likely due to morphological changes from thrombosis rather than the true disappearance of the lesion. Because ultrasonographic findings can change as thrombosis progresses, lesions that appear indistinct immediately before or after birth may become recognizable later, emphasizing the importance of careful evaluation. Taken together, when a fetal intra-abdominal cystic lesion with blood flow is identified, postnatal evaluation should not rely solely on the presence or absence of a cystic lesion. Assessment that considers the possibility of a thrombosed varix and screening for venous thrombosis, including the portal venous system, should be performed within the first one to two weeks of life.

In many reported cases of vitelline vein varix diagnosed postnatally, surgical intervention has been recommended [1,3-5,10]. However, in clinical practice, some cases have been managed conservatively with anticoagulation therapy, most commonly using heparin sodium, under close monitoring [13]. Further accumulation of cases and shared clinical experience is needed to clarify the indications for surgical intervention. This report provides a detailed correlation between prenatal imaging findings and the definitive intraoperative diagnosis, and demonstrates the clinical course of early postnatal thrombus formation. These findings provide useful guidance for diagnosis and management and enhance the understanding available in the limited existing literature.

## Conclusions

This report highlights that when a cystic intra-abdominal lesion with detectable blood flow is identified in a fetus, a vitelline vein varix should be considered in the differential diagnosis, particularly in cases detected relatively early (before the mid-second trimester), when the lesion exceeds 20 mm in diameter, and when an atypical venous course inconsistent with the normal umbilical vein pathway is observed. Careful evaluation of the detailed venous anatomy based on these ultrasonographic findings may contribute to more accurate prenatal diagnosis. Furthermore, as early postnatal thrombus formation was observed in this case, close follow-up is warranted when a vitelline vein varix is suspected, including not only immediate postnatal assessment but also re-evaluation within the first one to two weeks of life. By providing a detailed correlation between prenatal imaging findings and definitive intraoperative diagnosis, and by clarifying the clinical course of early thrombus formation, this report offers practical insights to the limited existing literature. Accumulation of additional cases is expected to help establish standardized diagnostic criteria and optimal perinatal management strategies for this rare condition.
